# Clinical risk factors to predict prognosis in wake-up stroke patients: A retrospective study

**DOI:** 10.1097/MD.0000000000040584

**Published:** 2024-11-15

**Authors:** Qiwu Xu, Miaomiao Hu, Guoxiang Tan, Yong Zhao, Hao Yin, Ting Ding, Ying Zhou

**Affiliations:** a Department of Neurology, Tongling Municipal Hospital, Tongling, Anhui Province, China; b Key Laboratory of Digital Technology in Medical Diagnostics of Zhejiang Province, Dian Diagnostics Group Co., Ltd., Hangzhou, Zhejiang Province, China.

**Keywords:** intravenous thrombolysis, multimodal magnetic resonance imaging, nomogram, prognosis, wake-up stroke

## Abstract

This study aimed to develop and validate a clinical risk model based on clinical factors to predict prognosis in patients with wake-up stroke (WUS) after multimodal magnetic resonance imaging combined with recombinant tissue plasminogen activator intravenous thrombolysis. The study enrolled 263 patients with WUS, who were divided into the training (n = 162) and validation cohorts (n = 101). In the training cohort, patients were stratified based on modified Rankin Scale (mRS) score at 90 days after thrombolysis, with mRS ≤ 2 indicating a good prognosis (n = 117), and mRS > 2 indicating a poor prognosis (n = 45). Multivariate regression analyses were employed to identify independent risk factors and develop clinical risk models. The performance and stability of the clinical risk model were evaluated using receiver operating characteristic analysis and Hosmer–Lemeshow test. The clinical risk nomogram was constructed based on this model, and evaluated using decision curve analyses. Patients with poor prognosis showed a higher proportion of hyperlipidemia and diabetes and showed a higher levels of National Institute of Health Stroke Scale (NIHSS) at admission, NIHSS at 24 hours, triglyceride, and total cholesterol. Diabetes (odds ratio [OR] = 3.823), hyperlipidemia (OR = 7.361), NIHSS at admission (OR = 5.399), NIHSS at 24 hours (OR = 2.869), triglyceride (OR = 13.790), and total cholesterol (OR = 9.719) were independent predictors of poor prognosis in patients with WUS. Hosmer–Lemeshow test showed that the clinical risk model had a good fit in the training (*χ*^2^ = 19.573, *P* = .726) and validation cohorts (*χ*^*2*^ = 19.573, *P* = .726). The clinical risk model had an area under the curve value of 0.929 (95% confidence interval, 0.886–0.978) in the training cohort and 0.948 (0.906–0.989) in the validation cohort. The decision curve analysis indicated clinical risk nomogram has application value. The clinical risk model can effectively predict WUS prognosis outcomes.

## 1. Introduction

Wake-up stroke (WUS) is a unique type of acute ischemic stroke, characterized by the sudden onset of symptoms at awakening in previously asymptomatic individuals.^[1]^ Due to the patient’s illness during sleep, the exact onset time of the disease is unknown, often resulting in exclusion from thrombolytic treatment and a missed opportunity for optimal intervention, ultimately leading to poor prognosis. Studies have demonstrated that multimodal imaging can accurately assess the onset time of patients with WUS and guide intravenous thrombolytic therapy.^[2]^ Diffusion-weighted imaging (DWI) is highly sensitive to early lesions, which can be detected as early as 2 hours after onset, while fluid-attenuated inversion recovery (FLAIR) typically shows abnormal signals at around 4.5 hours after onset. Therefore, when there is a mismatch between DWI and FLAIR findings, it may suggest an ischemic stroke onset time within 4.5 hours, and intravenous thrombolysis could be considered to improve patient prognosis.^[3,4]^ Research has demonstrated that intravenous thrombolysis with plasminogen activator (rt-PA) is the only safe and effective treatment for WUS patients within 4.5 hours of onset,^[5]^ thereby increasing the proportion of WUS patients receiving intravenous thrombolysis. However, certain patients present with a poor prognosis, and it is necessary to further clarify the factors associated with poor prognosis.

The objective of this study was to develop a risk prediction model that integrates demographic information and clinical factors, and to construct an intuitive predictive nomogram for the prognosis of WUS. Therefore, we analyzed clinical data from 214 WUS patients admitted to Tongling Municipal Hospital between January 2020 and December 2022, and developed a clinical risk model.

## 2. Materials and methods

### 2.1. Study population

A total of 324 patients were admitted to Tongling Municipal Hospital from January 2020 to December 2023, out of which 307 underwent multimodal magnetic resonance imaging (MRI) scans and received rt-PA thrombolytic therapy within 4.5 hours of diagnosis. The study commenced in January 2023, retrospectively encompassing patient populations from January 2020 to December 2022 as the training cohorts, and prospectively incorporating patient populations from January to December 2023 as the validation cohorts. The inclusion criteria were: (1) meeting diagnostic criteria in the Chinese Guidelines for the Diagnosis and Treatment of Acute Ischemic Stroke 2018; (2) patients exhibiting normal conditions before sleep but experiencing symptoms of acute ischemic stroke on awakening; (3) age was older than 18 years old; (4) multimodal MRI scans indicating a DWI-FLAIR mismatch.

The exclusion criteria were: (1) patients signed the consent to refuse thrombolytic therapy at admission or were transferred to another hospital; (2) death during the first 24 hours; (3) patients with significant cerebral disorders, such as cerebral hemorrhage, brain trauma, or brain tumors, or major infectious diseases; (4) individuals receiving oral non-vitamin K antagonist and oral anticoagulants (such as rivaroxaban, apixaban) for chronic atrial fibrillation within 48 hours; (5) patients with intracranial macrovascular disease undergoing endovascular treatment; (6) patients who have underwent craniotomy within the past year; (7) patients lost to follow-up within 90 days after thrombolysis; (8) patients with incomplete data. All patients had signed informed consent, which was approved by the Ethics Committee of TongLing Municipal Hospital (No. SY2019-6).

### 2.2. Study data

The demographic information and clinical data of the WUS patients were collected. Demographic information included personal details such as name, gender, age, alcohol consumption, smoking, diabetes, hypertension, coronary heart disease, and hyperlipidemia. The clinical data encompassed MRI imaging results and laboratory tests conducted at admission (HbA1c, total cholesterol [TC], triglyceride [TG], high density lipoprotein [HDL-C], low density lipoprotein [LDL-C]), National Institute of Health Stroke Scale (NIHSS) score (at admission, 24 hours after thrombolysis, and 90 days after thrombolysis), modified Rankin Scale (mRS) scores at 90 days after thrombolysis, hemorrhage after thrombolysis, and symptomatic cerebral hemorrhage after thrombolysis.

All patients diagnosed with WUS underwent a head multimodal MRI scan and received thrombolytic therapy within 35 minutes of admission. The DWI-FLAIR mismatch observed on the multimodal MRI refers to high signal intensity on DWI but no corresponding high signal intensity on FLAIR at the same location, and rt-PA was used for thrombolytic therapy at a standard dose of 0.9 mg/kg. Ten percent of the dose was administered intravenously within 1 minute, while the remaining portion was slowly infused over a period of 60 minutes. After 24 hours of thrombolytic therapy, the patient undergoes a computed tomography scan of the head to evaluate for post-thrombolysis hemorrhage.

The blood samples were collected from patients upon admission on an empty stomach using EDTA-2K anticoagulant tubes and subsequently stored at a temperature range of 2 °C to 8 °C. The concentration of HbA1c was measured using high-performance liquid chromatography (MQ-2000PT glycosylated hemoglobin analyzer, Shanghai Huizhong Medical Technology Co., Ltd.), with a normal reference range of 4% to 6%. The levels of TC, HDL-C, and LDL-C were quantified using a latex-enhanced immunoturbidimetric assay on the BECKMAN Coulter AU5800 instrument from the United States.

The NIHSS and mRS were utilized to assess patient prognosis. NIHSS was employed to evaluate neurological deficits at admission, 24 hours and 90 days after thrombolysis, in which 0 to 6 was defined as mild injury, 6 to 13 was defined as moderate injury, and more than 13 was defined as severe injury.^[6]^ After thrombolytic therapy, the NIHSS score at admission was compared to that at 24 hours after thrombolysis. A decrease of ≥8 points in the NIHSS score indicated improved neurological function, while an increase of ≥4 points suggested symptomatic cerebral hemorrhage following thrombolysis.^[7]^ Patients were followed up through outpatient reviews or telephone interviews at 90 days after thrombolysis, and their prognosis was evaluated using the mRS score. A good prognosis was defined as a mRS score of 0 to 2, while a poor prognosis was defined as a score of 3 to 6.^[8]^ Based on their mRS scores at 90 day after thrombolysis, patients were categorized into either the good or poor prognosis group.

### 2.3. Construction of clinical risk model

Multivariate logistic regression is a widely used statistical model for predicting clinical risk.^[9]^ All dependent variables were categorical, defined as either 0 or 1. The model first establishes the logistic regression equation and then calculates the *P*-value format. Formula 1 shows the regression equation:

$$\begin{array}{l} Logit\;(P)=\beta_{0}+\beta_{1}x_{1}+\beta_{2}x_{2}+\ldots +\beta_{n}x_{n} \end{array}$$ (1)

The value $P/\left(1+P\right)=e^{logit\left(P\right)}$, Logit (P) can be obtained from formula 1, and the formula for calculating the *P*-value is as follows:

$$\begin{array}{l} P=e^{logit\left(P\right)}/1+e^{logit\left(P\right)}\; \end{array}$$ (2)

### 2.4. Statistical analysis

Statistical analysis was performed using SPSS (version 24.0) and R software (version 3.6.3). Shapiro–Wilk was used to test the normal distribution of measurement data. The normal distribution was expressed as mean ± standard deviation, and the *t* test was used for pairwise comparison between groups. Skewed distribution data were expressed as median (interquartile range), and pairwise comparison between groups was conducted using Mann–Whitney *U* test. Count data were presented as [n (%)] and inter-group comparisons were conducted using the chi-square test. Multivariate (binary logistic) regression analyses were performed to identify independent risk factors for poor prognosis in WUS patients, with a logistic regression model constructed accordingly. The Hosmer–Lemeshow test and receiver operating characteristic (ROC) curve analysis were employed to evaluate the model’s goodness-of-fit and predictive accuracy, with the calculation of area under ROC curve (AUC), sensitivity, and specificity. A clinical risk nomogram was developed using the “rms” package in R software, and decision curve analysis (DCA) was conducted to assess the clinical usefulness of the nomogram. A *P*-value < .05 was considered significant in all analyses.

## 3. Results

### 3.1. Baseline information

A total of 307 patients were enrolled in the study, with 44 exclusions: 11 due to mortality within 24 hours, 10 transfers, 5 preexisting brain disease or major infectious illness, and 18 lost to follow-up within 3 months. According to the time of admission, patients were divided into training (n = 162) and validation (n = 101) cohorts. The training cohort consisted of 80 males and 82 females with an average age of (71.54 ± 6.58) years. The primary underlying conditions were diabetes (37.65%) and hypertension (96.30%), with 45 cases exhibiting a poor prognosis and 117 cases demonstrating a good prognosis. The validation cohort comprised 53 males and 48 females, with an average age of (69.81 ± 5.12) years. Among them, 27 cases presented a poor prognosis while 74 cases demonstrated a good prognosis. The demographic, clinical features, molecular and outcome variables for WUS patients in the training cohort were detailed in Table 1.

**Table 1:** Univariate analysis. Demographic, clinical features, molecular and outcome variables for WUS patients in the training cohort (n = 162).

Variables	Good prognosis (n = 117)	Poor prognosis (n = 45)	*P*-value
Demographic variables			
Age (years)	74.0 ± 5.81	72.58 ± 6.62	.313
Male	62 (52.99)	20 (44.45)	.330
Smoking	26 (22.22)	9 (20.00)	.758
Alcohol consumption	32 (27.35)	11 (24.45)	.708
Diabetes	29 (24.79)	32 (71.11)	<.001
Hypertension	111 (94.87)	45 (100.00)	.122
Coronary disease	29 (24.79)	16 (35.56)	.17
Hyperlipidemia	17 (14.53)	19 (42.22)	<.001
Clinical features			
NIHSS (at admission)			<.001
> 13	18 (15.38)	35 (77.78)	
≤ 13	99 (84.62)	10 (22.22)	
NIHSS (at 24 hours)			<.001
≥ 6	23 (19.66)	32 (71.11)	
< 6	94 (80.34)	13 (28.89)	
Bleeding after thrombolysis	1 (0.85)	3 (6.67)	.033
Symptomatic bleeding	0 (0)	2 (4.45)	.022
Molecular			
HbA1c (%)	5.95 ± 1.40	6.26 ± 1.51	.141
TG (mmol/L)	3.05 ± 1.36	4.73 ± 1.53	<.001
TC (mmol/L)	4.63 ± 1.15	5.09 ± 1.31	.034
HDL-C (mmol/L)	1.46 ± 0.35	1.42 ± 0.36	.426
LDL-C (mmol/L)	3.09 ± 1.34	3.62 ± 1.45	.055
Hcy (µmol/L)	27.39 ± 25.01	25.01 ± 2.39	.678
Outcome			
NIHSS (at 3 months)	2 [1, 2]	4 [2, 5]	<.001
mRS (at 3 months)	1 [1, 1]	3 [3, 4]	<.001

Data presented as n (%), mean ± standard deviation or median (interquartile range). Hcy = homocysteine, HDL-C = high density lipoprotein, LDL-C = low density lipoprotein, mRS = modified Rankin Scale score, NIHSS = National Institutes of Health Stroke Scale score, TC = total cholesterol, TG = triglyceride.

### 3.2. Demographic information and clinical features

**Figure 1.**
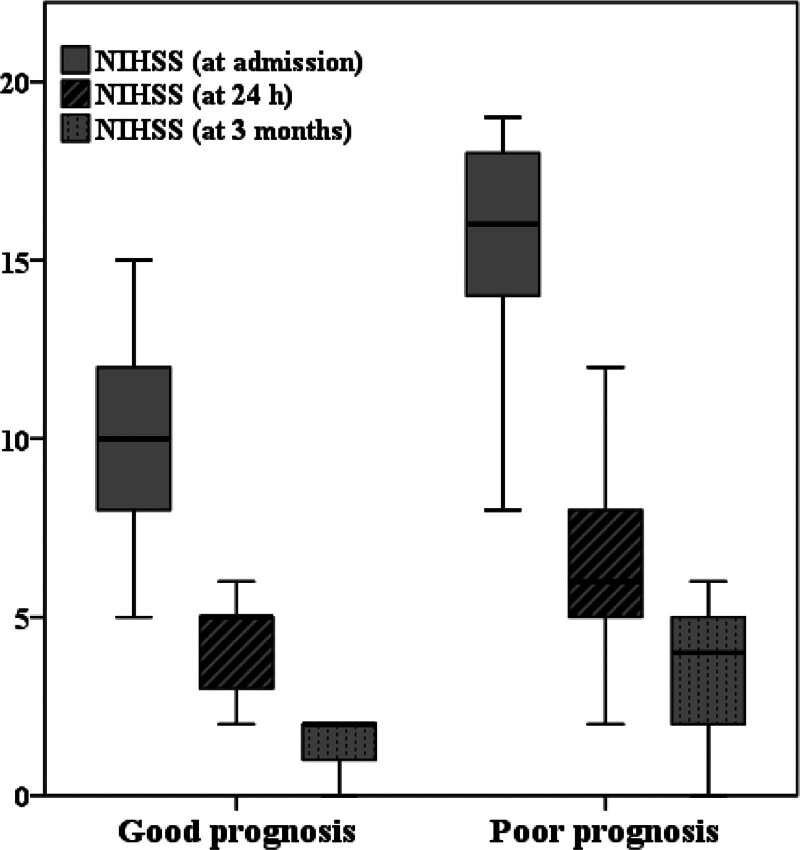
NIHSS evolution of good prognosis and poor prognosis. NIHSS = National Institute of Health Stroke Scale.

Patients with a poor prognosis exhibited a higher prevalence of hyperlipidemia and diabetes, while no significant differences were observed in terms of gender, age, smoking, alcohol consumption, or other underlying disease risk factors between the 2 groups (Table 1). Based on clinical features, patients with a poor prognosis presented with higher NIHSS scores at admission (*P* < .001) and NIHSS at 24 hours (*P* = .002). Patients with a poor prognosis exhibited a higher proportion of NIHSS scores >13 at admission and NIHSS scores >6 at 24 hours. Additionally, no significant differences were observed in bleeding after thrombolysis or symptomatic bleeding. Table S1, Supplemental Digital Content, http://links.lww.com/MD/N975 and Figure 1 detail the NIHSS evolution at different time-points between patients with good and poor prognoses. While both groups experienced a decline in NIHSS scores at different time-points, patients with poor prognoses exhibited higher neurological deficits at each time point.

### 3.3. Molecular and outcome variables

**Figure 2.**
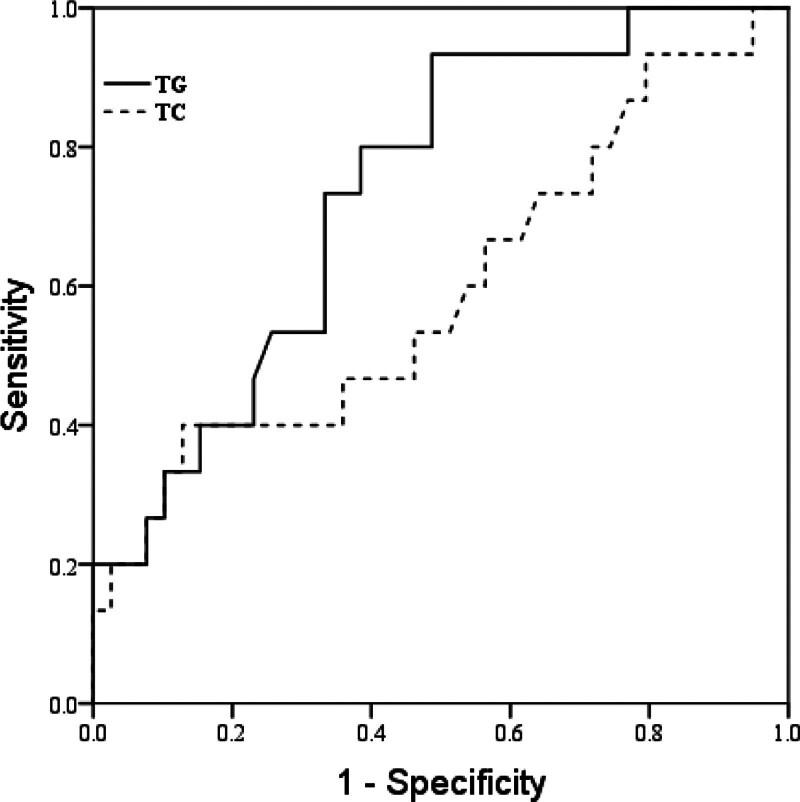
ROC curve analysis to establish the sensitivity and specificity of TC, TG levels to predict poor prognosis risk. ROC = receiver operating characteristic, TC = total cholesterol, TG = triglyceride.

Molecular markers analysis revealed that patients with poor prognosis exhibited elevated levels of TG and TC. Conversely, no significant differences were observed in glycemic variables (HbA1c), lipid variables (HDL-C and LDL-C), or cardiovascular disease markers. We investigated the predictive efficacy of TC and TG levels in identifying poor prognosis. The ROC curve analysis revealed that the AUC for TC and TG was 0.594 and 0.738, respectively (Fig. 2). For a cutoff point of 5.91 mmol/L for TC levels, the sensitivity and specificity were 86.7% and 87.2%, respectively. For a cutoff point of 2.78 mmol/L for TG levels, the sensitivity and specificity were 93.3% and 58.7%, respectively. Additionally, patients with poor prognosis demonstrated higher scores on NIHSS and mRS at 3 months.

### 3.4. Independent risk factor analysis

From the multivariate logistic regression analysis, we have identified several independent factors that were associated with poor prognosis, including diabetes (odds ratio [OR] = 3.823), hyperlipidemia (OR = 7.361), NIHSS at admission (OR = 5.399), NIHSS at 24 hours (OR = 2.869), TG (OR = 13.790), and TC (OR = 9.719). The independent risk factors included diabetes, hyperlipidemia, NIHSS (at admission), NIHSS (at 24 hours), TG, and TC (Table 2). A clinical risk prediction model was established using multivariate logistic regression.

**Table 2:** Multivariate logistic regression analysis of factors related to poor prognosis risk.

Factor	Assignment	OR (95% CI)	*P*-value
Diabetes	Yes = 1, no = 0	3.823 (1.037–14.088)	.044
Hyperlipidemia	Yes = 1, no = 0	7.361 (1.600–33.863)	.010
NIHSS (at admission)	Score > 13 = 1, Score ≤ 13 = 0	5.399 (1.567–26.934)	<.001
NIHSS (at 24 hours)	Score ≥ 6 = 1, Score < 6 = 0	2.869 (0.778–10.586)	.014
TG	Level ≥ 2.78 mmol/L = 1, Level < 2.78 mmol/L = 0	13.790 (2.311–82.295)	.004
TC	Level ≥ 5.91 mmol/L = 1, Level < 5.91 mmol/L = 0	9.719 (1.677–56.341)	.011

CI = confidence interval, NIHSS = National Institutes of Health Stroke Scale score, OR = odds ratio, TC = total cholesterol, TG = triglyceride.

### 3.5. Establishment and performance of the clinical risk model

**Figure 3.**
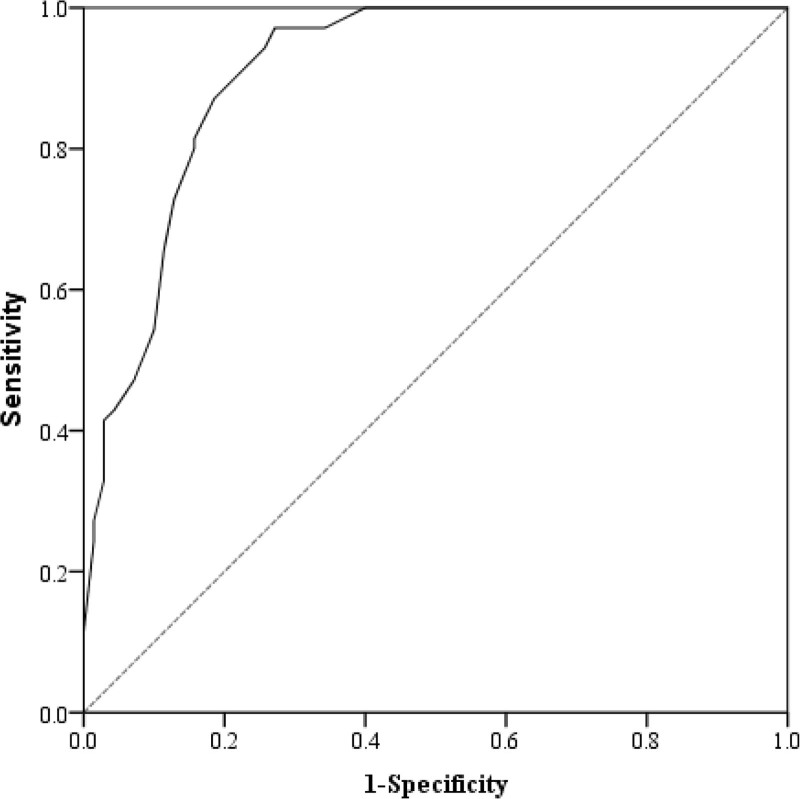
ROC curves of the clinical risk model in the training cohort. ROC = receiver operating characteristic.

**Figure 4.**
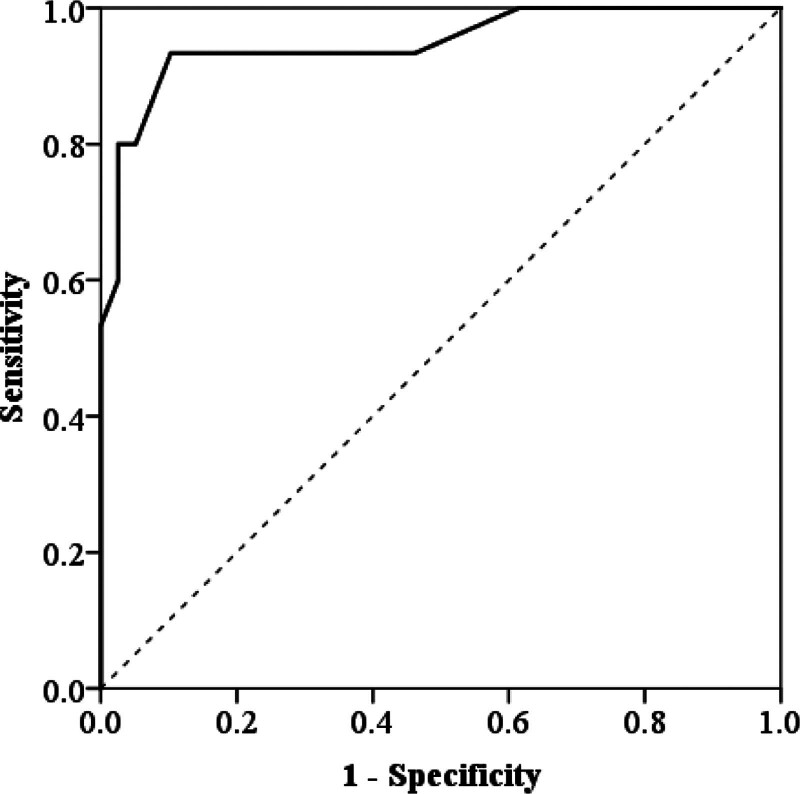
ROC curve of the clinical risk model in the validation cohort. ROC = receiver operating characteristic.

Six factors associated with poor prognosis were identified through multivariate logistic regression analysis, and these factors were used as predictive variables to construct a clinical risk model. The model can be expressed as *P* = 1/[1 + exp (6.477 − 1.341 × diabetes − 1.996 × hyperlipidemia − 3.978 × NIHSS at admission − 1.054 × NIHSS at 24 hours − 2.624 × TG − 2.274 × TC)], where *P* represents the predicted risk probability by the model. Hosmer–Lemeshow test showed that the clinical risk model had a good fit in the training (*χ*^2^ = 19.573, *P* = .726) and validation cohorts (*χ*^2^ = 19.573, *P* = .726). The clinical risk model showed an AUC of 0.929 (95% confidence interval [CI], 0.886–0.978), with a sensitivity of 95.1% and specificity of 88.9% in the training cohort (Fig. 3). In the validation cohort, the model achieved an AUC of 0.948 (95% CI, 0.906–0.989) with a sensitivity and specificity of 93.3% and 89.7%, respectively (Fig. 4).

**Figure 5.**
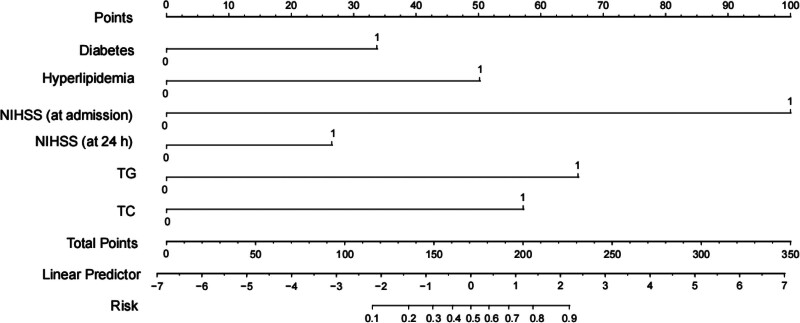
The nomogram based on clinical risk model and decision curve analysis. The developed nomogram based on clinical risk model to predict the risk of poor prognosis. Diabetes: 0, no diabetes; 1, diabetes. Hyperlipidemia: 0, no hyperlipidemia; 1, hyperlipidemia. NIHSS (at admission): 0, ≤13; 1, >13. NIHSS (at 24 hours): 0, <6; 1, ≥6. TG: 0, <2.775; 1, ≥2.775. TG: 0, <5.91; 1, ≥5.91. NIHSS = National Institutes of Health Stroke Scale score, TG = triglyceride, TC = total cholesterol.

**Figure 6.**
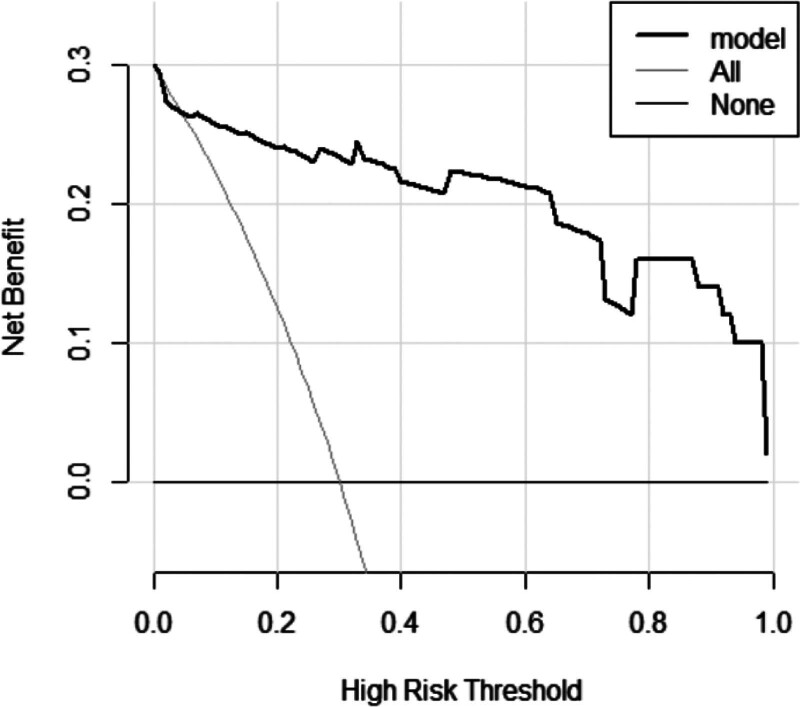
Decision curve analysis for the nomogram. The black line represents the net benefit of assuming no wake-up stroke (WUS) patients have poor prognosis. The gray line is the net benefit of assuming all WUS patients have poor prognosis. The bold black line represent the expected net benefit of predicting WUS prognosis outcome using the clinical risk model.

A nomogram was constructed based on this model, as depicted in Figure 5. The nomogram assigns a score to each factor, and the cumulative score of all factors corresponds to the risk of poor prognosis. DCA indicated that when the threshold was set at approximately 0.06 to 1, the nomogram model provided a more intuitive expression of prognostic outcomes than the clinical risk model (Fig. 6).

## 4. Discussion

In this study, demographic information, clinical features, outcome variables, and molecular markers associated with glucose metabolism, lipid profile, and cardiovascular dysfunction were assessed to confirm the risk factors for poor prognosis in WUS patients. A clinical risk model was developed based on identified risk factors, and a nomogram was utilized to optimize clinical management and treatment strategies. A total of 263 patients diagnosed with WUS were included in this study, all of whom received intravenous rt-PA thrombolytic therapy guided by multimodal MRI. Significant differences in demographics, clinical features, and molecular markers were observed between the good prognosis group and the poor prognosis group among WUS patients in the training cohort. Consistent with previous research,^[10,11]^ it can be inferred that individuals in the poor prognosis group had a higher prevalence of diabetes and hyperlipidemia, while their hypertension prevalence was comparable to that of those in the good prognosis group. The study demonstrated a positive correlation between higher NIHSS scores at admission and at 24 hours of thrombolysis, and poorer patient prognosis, which is consistent with the findings of this investigation.^[12]^ We can say that the poor prognosis group presented with more severe symptoms at admission, however, their NIHSS score evolution was consistent with that of the good prognosis group.

Although imaging studies are valuable for diagnosing WUS, there remains a lack of early predictors to identify patients with poor prognosis.^[13]^ Our findings indicate that the poor prognosis of WUS patients is linked to lipid molecular markers, specifically TC and TG. It is widely recognized that elevated levels of TC and TG are associated with the development of atherosclerosis,^[14]^ cardiovascular disease,^[15]^ and poor prognosis of stroke.^[16]^ Clinical investigations have demonstrated that elevated concentrations of TC and TG are associated with an increased risk of stroke, as well as poorer prognosis in terms of stroke recovery and mortality rates.^[17]^ Therefore, TC and TG were utilized as early predictors to assess the efficacy of predicting poor prognosis in WUS patients, and the final AUC were 0.594 and 0.738, respectively. An AUC >0.7 indicated better predictive efficiency, demonstrating their significant predictive value.

Multivariate logistic regression analysis revealed that diabetes, hyperlipidemia, NIHSS at admission, NIHSS at 24 hours, TG, and TC were independent risk factors associated with poor prognosis in WUS patients. Compared to other clinical factors, NIHSS at admission, TC, and TG have a more significant impact on poor prognosis. Prolonged hyperglycemia in diabetic patient leads to endothelial cell damage and increased thrombosis. Moreover, the binding of glycosylated hemoglobin to its receptor activates coagulation factors, resulting in vascular contraction and further disease progression. This may even lead to reocclusion after thrombolysis.^[18,19]^ Hyperlipidemia can result in metabolic disorders and abnormalities, such as platelet adhesion, aggregation, and vasodilator hormone release. These factors contribute to faster thrombosis after stroke and more challenging thrombolysis.^[20]^ The NIHSS score serves as a clinical indicator of the degree of neurological impairment in stroke patients and is positively correlated with disease severity.^[21]^ Therefore, NIHSS score at admission and at 24 hours after thrombolysis NIHSS scores are predictive of poor prognosis.

The risk model of this study incorporated more clinical factors, such as smoking, alcohol consumption, and diabetes, and developed a corresponding nomogram to visually represent the risk factors more comprehensively. The clinical risk model in this study demonstrated a high predictive value with an AUC value of 0.929 (95% CI, 0.886–0.978) in the training cohort and 0.948 (0.906–0.989) in the validation cohort. A nomogram depicts the correlation between each variable, with each variable corresponding to a line segment whose length reflects its contribution to the outcome, while the scale represents the value range.^[22]^ We developed a novel nomogram based on the clinical risk model, and the DCA demonstrated that our nomogram model provided a more intuitive representation of the prognostic outcome for WUS patients compared to the clinical risk model.

Our study has certain limitations. Firstly, it is a retrospective, single-center study primarily conducted in the Tongling region of Anhui Province, China. The restricted geographical scope inherent to this type of investigation may potentially impact the generalizability of our findings. Secondly, WUS is frequently accompanied by comorbidities such as hyperlipidemia, hypertension, and diabetes mellitus. Distinct characteristics associated with blood parameter values may be observed in different subtypes of WUS patients. However, we did not further stratify and compare WUS patients based on different TOAST classifications in this study, which could potentially affect the generalizability and applicability of our findings. Moreover, the limited number of WUS patients with poor prognosis in this study may impact the precision and clinical applicability of our model. Simultaneously, the small sample size also hinders us from mitigating potential selection bias. Furthermore, our future plans involve expanding the sample size and stratifying WUS patients based on subtypes, while also incorporating MRI image-related factors to augment the overall performance level of the risk model.

## 5. Conclusion

In conclusion, the clinical risk model effectively predicted the prognosis of WUS patients for 3 months following intravenous thrombolysis. The adjunctive clinician promptly modified the appropriate therapeutic approach during the initial phases of treatment, which could potentially result in a substantial enhancement in the poor prognosis for WUS patients.

## Acknowledgments

We are grateful for all the staff at the medical records department for their help in data collection.

## Author contributions

**Conceptualization:** Qiwu Xu.

**Data curation:** Miaomiao Hu.

**Investigation:** Guoxiang Tan.

**Methodology:** Qiwu Xu.

**Resources:** Hao Yin.

**Validation:** Yong Zhao, Ting Ding.

**Visualization:** Ying Zhou.

**Writing – original draft:** Miaomiao Hu.

**Writing – review & editing:** Qiwu Xu.

## Supplementary Material
