# Supplementary material for: Clinical risk factors to predict prognosis in wake-up stroke patients: A retrospective study
Source: Medicine (Baltimore). 2024 Nov 15;103(46):e40584. doi: 10.1097/MD.0000000000040584 (PMC11575979; doi:10.1097/MD.0000000000040584)
Supplement: Supplementary file 1 [file medi-103-e40584-s001.doc]

**Supplementary Table 1. NIHSS evolution of good prognosis and poor prognosis**

| Group | NIHSS  (at admission) | NIHSS  (at 24 h) | NIHSS  (at 3 months) | *P* | *H* |
| --- | --- | --- | --- | --- | --- |
| Good prognosis  (n = 117) | 10 [8, 12] | 5 [3, 5] | 2 [1, 2] | < 0.001 | 99.153 |
| Poor prognosis  (n = 45) | 16 [12, 18] | 6 [5, 8] | 4 [2, 5] | < 0.001 | 24.813 |

Data presented as median (interquartile range). NIHSS = National Institutes of Health Stroke Scale score.
